# Supplementary material for: Phospho‐regulated Bim1/EB1 interactions trigger Dam1c ring assembly at the budding yeast outer kinetochore
Source: EMBO J. 2021 Jul 27;40(18):e108004. doi: 10.15252/embj.2021108004 (PMC8441410; doi:10.15252/embj.2021108004)
Supplement: Supplementary file 2 — Appendix [file EMBJ-40-e108004-s001.pdf]

Appendix for "Phospho-regulated Bim1/EB1 interactions trigger Dam1c ring assembly at the budding yeast outer kinetochore" by Alexander Dudziak, Lena Engelhard, Cole Bourque, Björn Udo Klink, Pascaline Rombaut, Nikolay Kornakov, Karolin Jänen, Franz Herzog, Christos Gatsogiannis and Stefan Westermann

Table of contents

Appendix Figure S1 2

Appendix Figure S1 – Figure Legend 3

Appendix Figure S2 4

Appendix Figure S2 – Figure Legend 4

Appendix Figure S3 5

Appendix Figure S3 – Figure Legend 5

Appendix Table S1 6

Appendix Table S2 7

Appendix Table S3 8

Appendix Figure S1

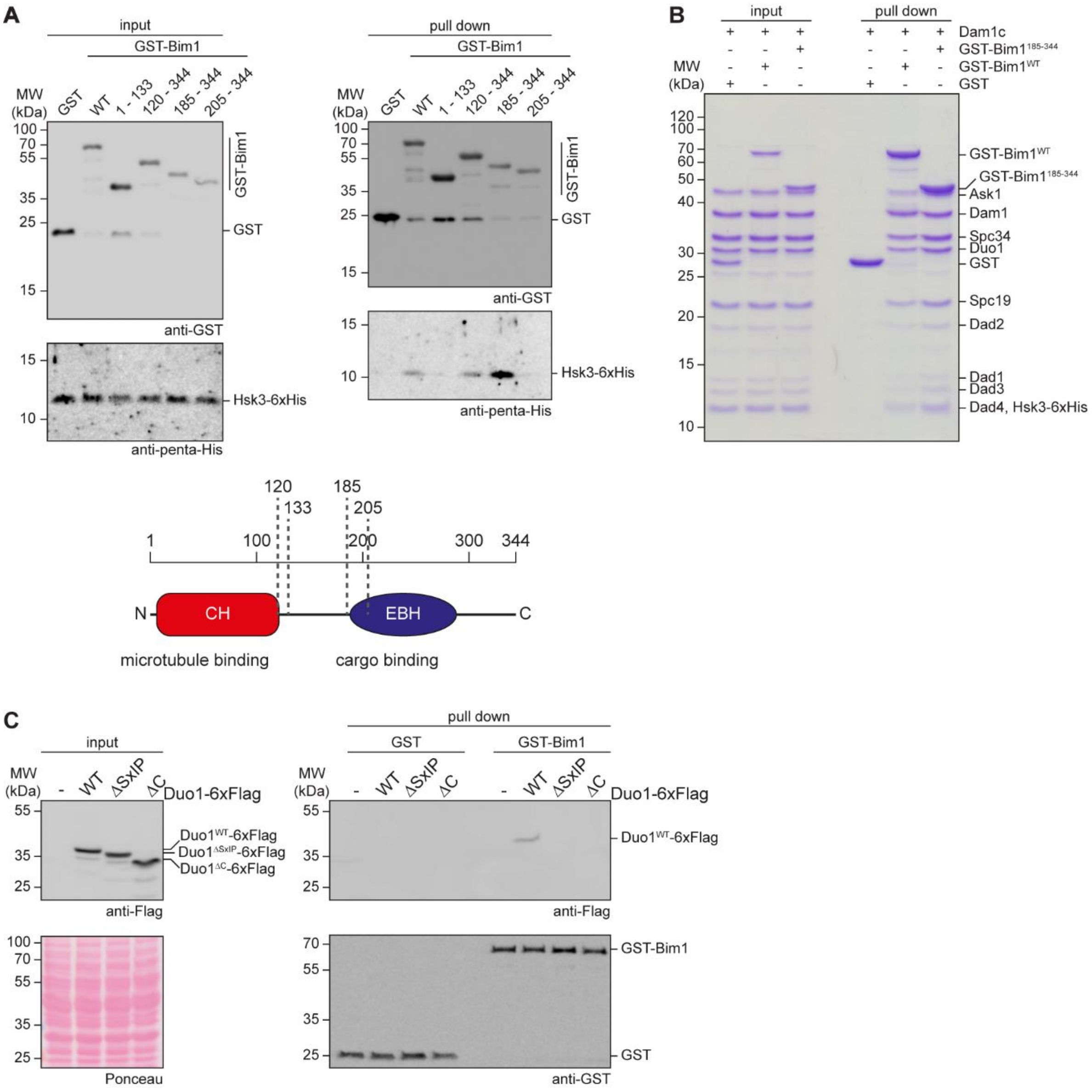

Appendix Figure S1 – Figure Legend

Formation of the Dam1c-Bim1 complex is mediated by an SxIP-EBH interaction

A: Recombinant GST fusions of truncated Bim1 were immobilized on beads and incubated with recombinant Dam1^{WT}c. Input and pull down samples were analyzed by western blot (top). Fusion proteins comprised the CH domain (1 – 133), linker and EBH domain (120 – 344), EBH domain (185 – 344) or an N-terminally truncated EBH domain (205 – 344). An illustration of Bim1 highlighting the relevant domains is depicted at the bottom. For reasons of simplicity, Bim1 is shown as monomer. CH: calponin homology, EBH: end-binding homology.

B: Pull down assay as described in A. GST, GST-Bim1^{WT} and GST-Bim1$^{185-344}$ were immobilized on beads and incubated with Dam1c. Input and pull down samples were analyzed by SDS-PAGE and Coomassie staining.

C: Pull down assay with cell lysates from yeast strains expressing different 6xFlag-tagged Duo1 alleles. Immobilized GST-Bim1 was incubated with soluble cell lysates and binding of Duo1-6xFlag was analyzed by western blot analysis of pull down samples. Duo1$^{\Delta C}$ is an R223Stop mutation which truncates Duo1 immediately in front of the SxIP motif.

Appendix Figure S2

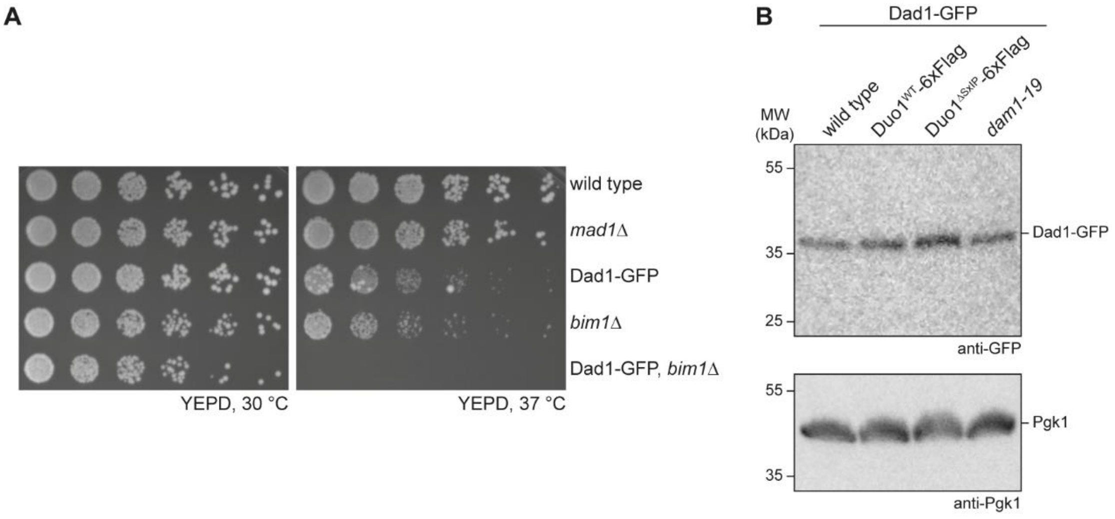

Appendix Figure S2 – Figure Legend

Further analysis of various Dad1-GFP strains

A: Serial dilution assay of Dad1-GFP and *bim1*Δ strains. Cells were spotted as serial dilution on YEPD plates and incubated at 30 °C and 37 °C.

B: Western blot analysis of cellular Dad1-GFP levels in strains with different Duo1 and Dam1 alleles. Pgk1 was used as loading control.

Appendix Figure S3

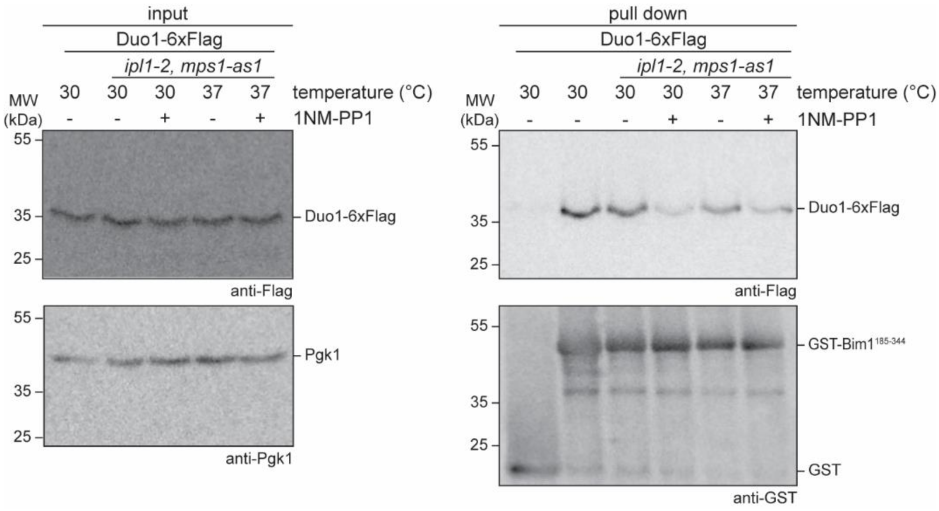

Appendix Figure S3 – Figure Legend

Inhibition of Mps1 impairs Dam1c-Bim1 binding

A strain carrying both the analog sensitive *mps1-as1* and temperature sensitive *ipl1-2* alleles was grown under the indicated conditions (30 °C or 37 °C, in the absence or presence of 10 µM 1NM-PP1). A strain with wild type alleles of both kinases was used as control. Binding of Dam1c to immobilized GST-Bim1$^{185\text{-}344}$ was analyzed in a pull down assay. Pgk1 serves as loading control.

Appendix Table S1

Protein	Position	Position in Bim1
Spc34	K46	K110
Spc34	K46	K134
Spc34	K46	K145
Spc34	K70	K145
Spc34	K169	K145
Spc34	K249	K145
Spc34	K274	K145
Dam1	K252	K110
Dam1	K252	K145
Dam1	K307	K110
Dam1	K307	K223
Dam1	K320	K110
Dam1	K320	K145
Spc19	K79	K110
Spc19	K79	K122
Spc19	K79	K145
Dad4	K21	K145
Duo1	K236	K223

Appendix Table S1 List of crosslinks between Bim1 and subunits of Dam1c.

Appendix Table S2

Protein	Position	Position in Duo1
Spc34	K70	K33
Spc34	K94	K33
Spc34	K70	K36
Spc34	K46	K215
Spc34	K46	K236
Spc34	K65	K236
Spc34	K70	K236
Spc34	K126	K236
Spc19	K79	K183
Spc19	K76	K236

Appendix Table S2 List of crosslinks between Duo1 and Spc19 and Spc34 in the presence of Bim1.

Appendix Table S3

Position in Dam1	Position in Duo1
K252	K169
K252	K177
K252	K183
K252	K215
K252	K236
K256	K131
K256	K169
K256	K236
K307	K169
K307	K183
K307	K215
K307	K236
K320	K169
K320	K174
K320	K215
K320	K236

Appendix Table S3 List of identified crosslinks between Duo1 and the C-terminus of Dam1. Only crosslinks involving lysine residues which are not present in *dam1-19* (Q205Stop) are listed.
