# Supplementary material for: Phospho‐regulated Bim1/EB1 interactions trigger Dam1c ring assembly at the budding yeast outer kinetochore
Source: EMBO J. 2021 Jul 27;40(18):e108004. doi: 10.15252/embj.2021108004 (PMC8441410; doi:10.15252/embj.2021108004)
Supplement: Supplementary file 3 — Expanded View Figures PDF [file EMBJ-40-e108004-s005.pdf]

Expanded View Figures

Figure EV1. Additional characterization of Dam1c interactions.

A Binding of Stu2 to Dam1c was analyzed by analytical size-exclusion chromatography. Chromatograms and SDS–PAGE analysis of elution fractions are shown. The large peak at an elution volume of 2 ml is caused by the 3xFlag peptide used for elution of Stu2-Flag during purification.

B Pull-down assay with immobilized Stu2-Flag. Stu2-flag from Sf9 cells was immobilized on beads and incubated with either, buffer, Dam1c or Bik1. Empty beads were used as control for unspecific binding of proteins to beads. Input and pull-down samples were analyzed by SDS–PAGE. HC and LC denote the heavy and light chain of the anti-Flag antibody, respectively. Subunits of Dam1c are labeled in blue. Bik1 and Stu2-Flag are labeled in green and orange, respectively.

C Lysine–lysine proximity map of Dam1c crosslinked with BS3 in the absence of Bim1. Each subunit of the complex is represented by a blue bar. Blue and red lines indicate intermolecular and intramolecular crosslinks, respectively.

D, E Representative TEM micrographs of negatively stained Dam1^{WT}c (D) and Dam1^{WT}c-Bim1 complex (E) (scale bar: 100 nm). Subsequent 2D classification of Dam1^{WT}c (D) revealed a mixture of Dam1c monomers (inset, upper row) and dimers (inset, lower row), whereas 2D classification of crosslinked Dam1^{WT}c-Bim1 complex (E) revealed monodispersed particles of Dam1c-dimers + Bim1 (inset). Most particles adopt a side-view orientation on the EM grid. To allow for a direct structural comparison, the monomers were excluded from further analysis after this initial round of 2D classification.

 © 2021 The Authors

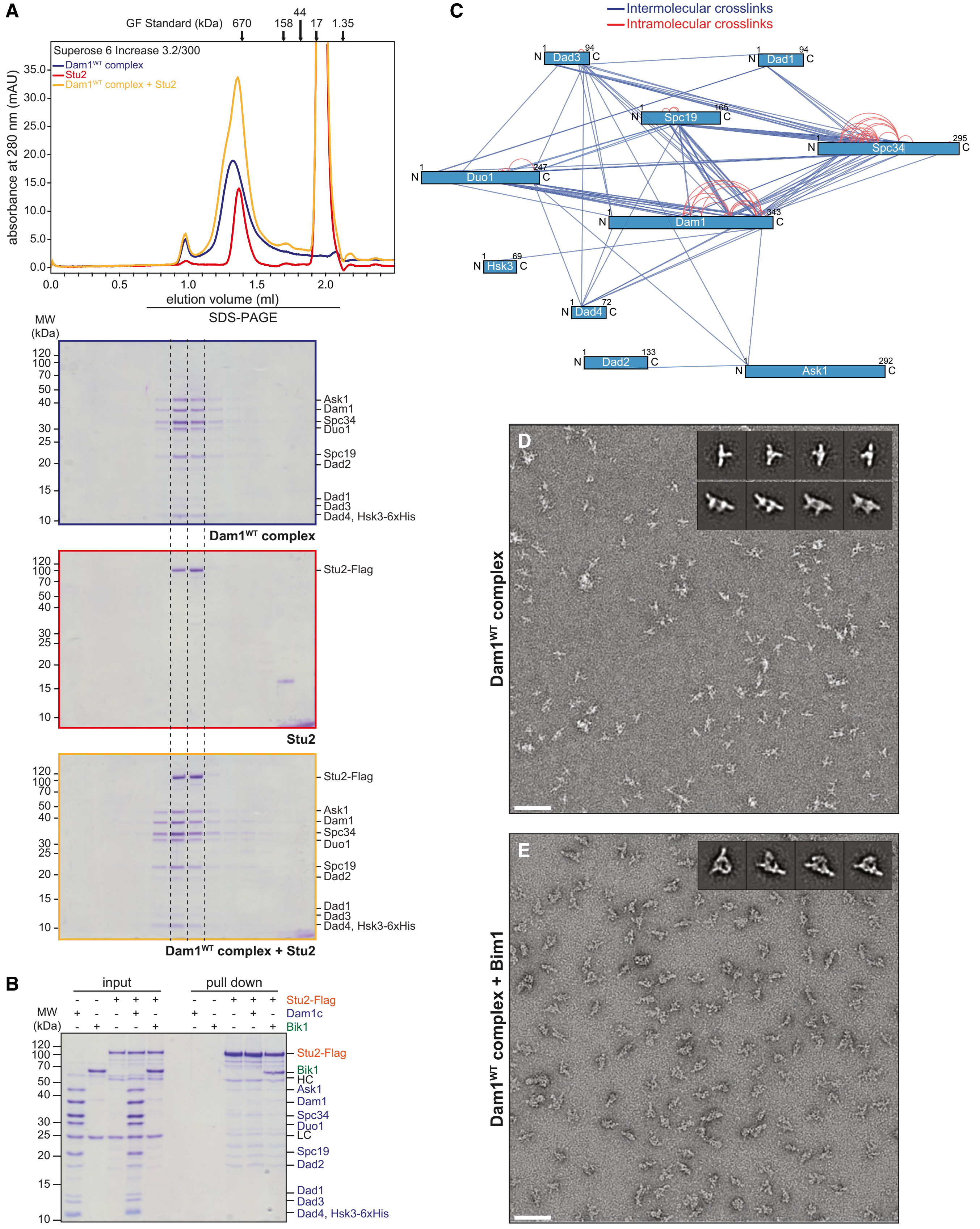
A
GF Standard (kDa) 670 158 44 17 1.35
Superose 6 Increase 3.2/300
Dam1WT complex
Stu2
Dam1WT complex + Stu2
absorbance at 280 nm (mAU)
elution volume (ml)
SDS-PAGE
MW (kDa)
Ask1
Dam1
Spc34
Duo1
Spc19
Dad2
Dad1
Dad3
Dad4, Hsk3-6xHis
Dam1WT complex
Stu2-Flag
Stu2
Dam1WT complex + Stu2
B
input
pull down
Stu2-Flag
Dam1c
Bik1
Bik1
HC
LC
C
Intermolecular crosslinks
Intramolecular crosslinks
Dad3
Dad1
Spc19
Spc34
Duo1
Dam1
Hsk3
Dad4
Dad2
Ask1
D
Dam1WT complex
E
Dam1WT complex + Bim1

Figure EV1.

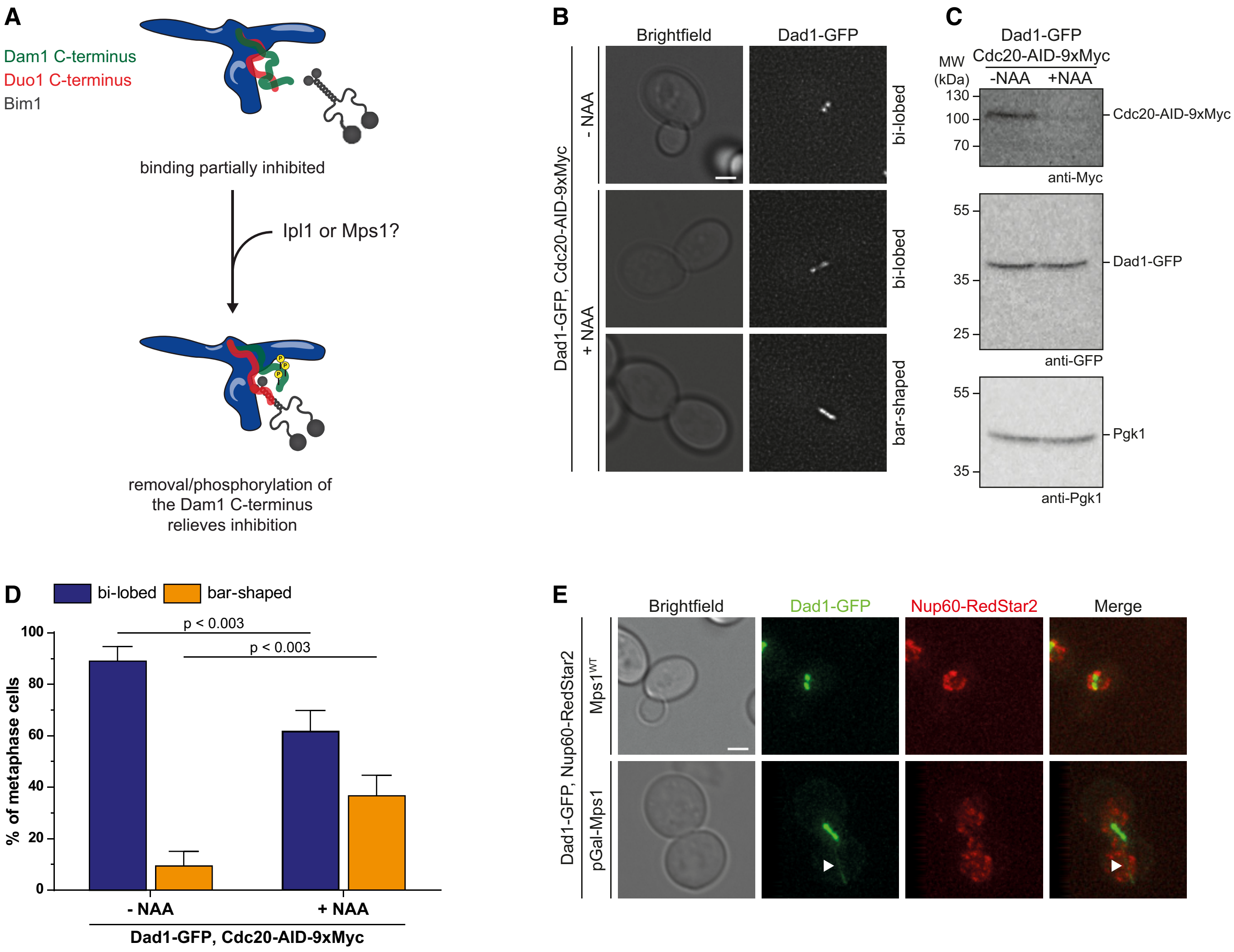

Figure EV2. Detailed live cell microscopy analysis of Dad1-GFP localization during metaphase arrest.

A Model of the intramolecular inhibition of the Duo1 SxIP motif by the Dam1 C-terminus. The unstructured C-terminus of Dam1 partially binds to the Duo1 C-terminus, which contains the SxIP motif required for Bim1 binding. Thus, binding of Bim1 is inhibited. Removal of the Dam1 C-terminus either by truncation or by phosphorylation relieves the intramolecular inhibition and allows binding of Bim1 with high affinity.

B Live cell microscopy of Cdc20-AID-9xMyc strain in the absence or presence of the auxin analog NAA. Addition of NAA rapidly induces proteasomal degradation of Cdc20 resulting in a metaphase arrest. Cells arrested in metaphase show either a bi-lobed or a bar-shaped localization of Dad1-GFP. Scale bar: 2 μm.

C Western blot showing the degradation of Cdc20-AID-9xMyc after addition of NAA. Cellular amounts of Dad1-GFP were not affected by NAA treatment. Pgk1 served as loading control.

D Quantification of bi-lobed and bar-shaped Dad1-GFP clusters in Cdc20-AID-9xmyc cells without (left) or after treatment with NAA (right). Exemplary images of the respective categories are depicted in (B). Mean and standard deviation from three independent experiments are shown. A two-way ANOVA test with Sidak's test for multiple comparisons was used for calculation of *P*-values.

E Comparison of Dad1-GFP and Nup60-RedStar2 localization. Extended microtubules formed during overexpression of Mps1 are decorated with Dad1-GFP. Nup60-RedStar2 marks the shape of the nucleus. White arrowheads label a microtubule extension. Scale bar: 2 μm.

 © 2021 The Authors

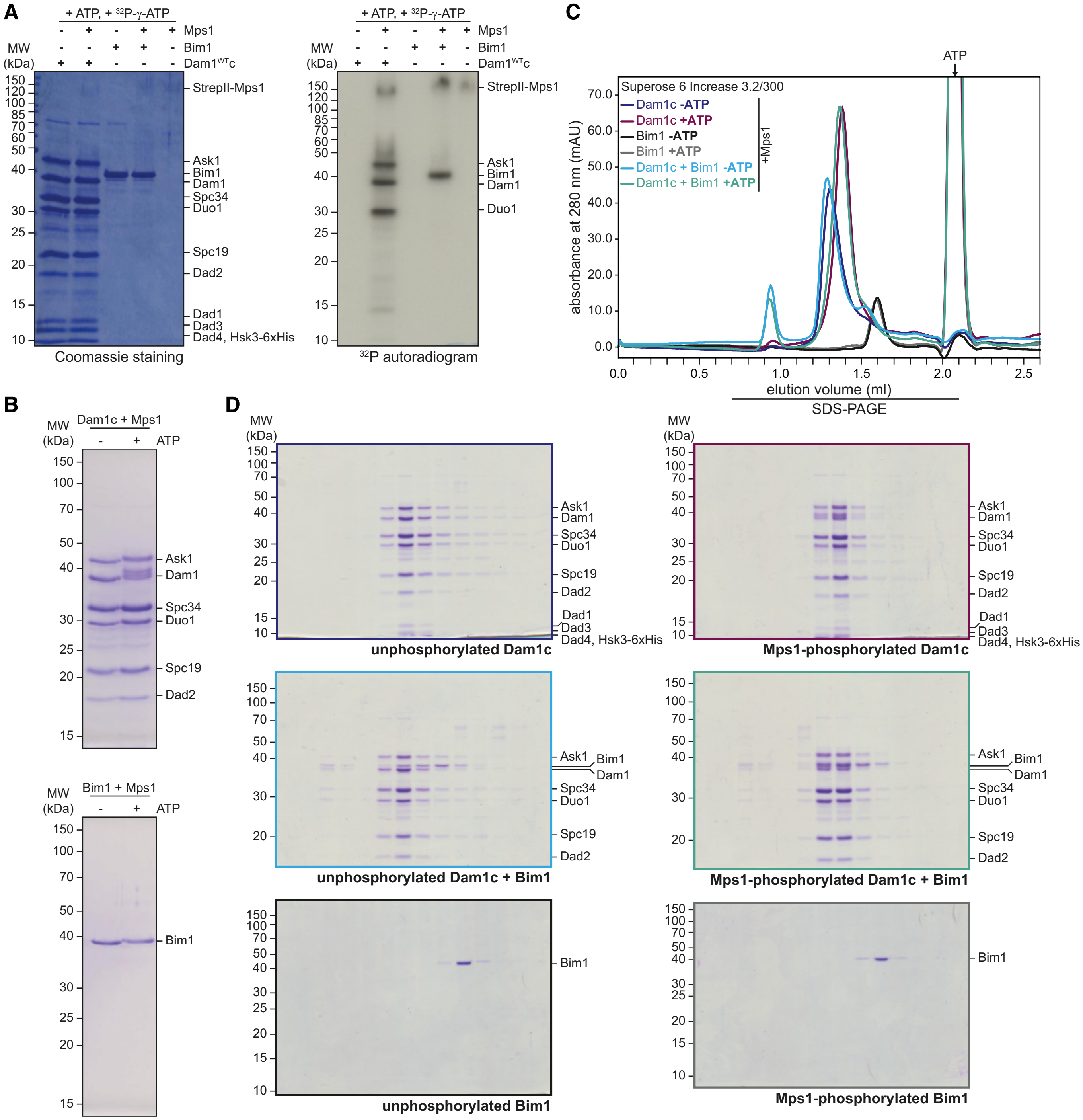

Figure EV3. Identification of Ask1, Duo1, and Bim1 as novel Mps1 substrates.

A *In vitro* phosphorylation of Dam1c and Bim1 by Mps1. Dam1c and Bim1 were incubated with Mps1 in the presence of ^{32}P-γ-ATP. Samples were analyzed by SDS–PAGE, and proteins were stained with Coomassie. Incorporation of ^{32}P was detected by autoradiography.

B SDS–PAGE analysis of unphosphorylated and Mps1-phosphorylated Dam1c (top) and Bim1 (bottom). Ask1, Dam1, Duo1, and Bim1 show reduced mobility after phosphorylation.

C, D Size-exclusion chromatography of Dam1c and Bim1 after phosphorylation by Mps1. Recombinant Dam1c, Bim1, or Dam1c and Bim1 was incubated with Mps1 in the absence or presence of ATP. Samples were subjected to size-exclusion chromatography with a buffer containing 400 mM NaCl, and elution fractions were analyzed by SDS–PAGE. Chromatograms are shown in (C). SDS–PAGE analysis of elution fractions of unphosphorylated (left) and phosphorylated samples (right) is depicted in (D).

© 2021 The Authors

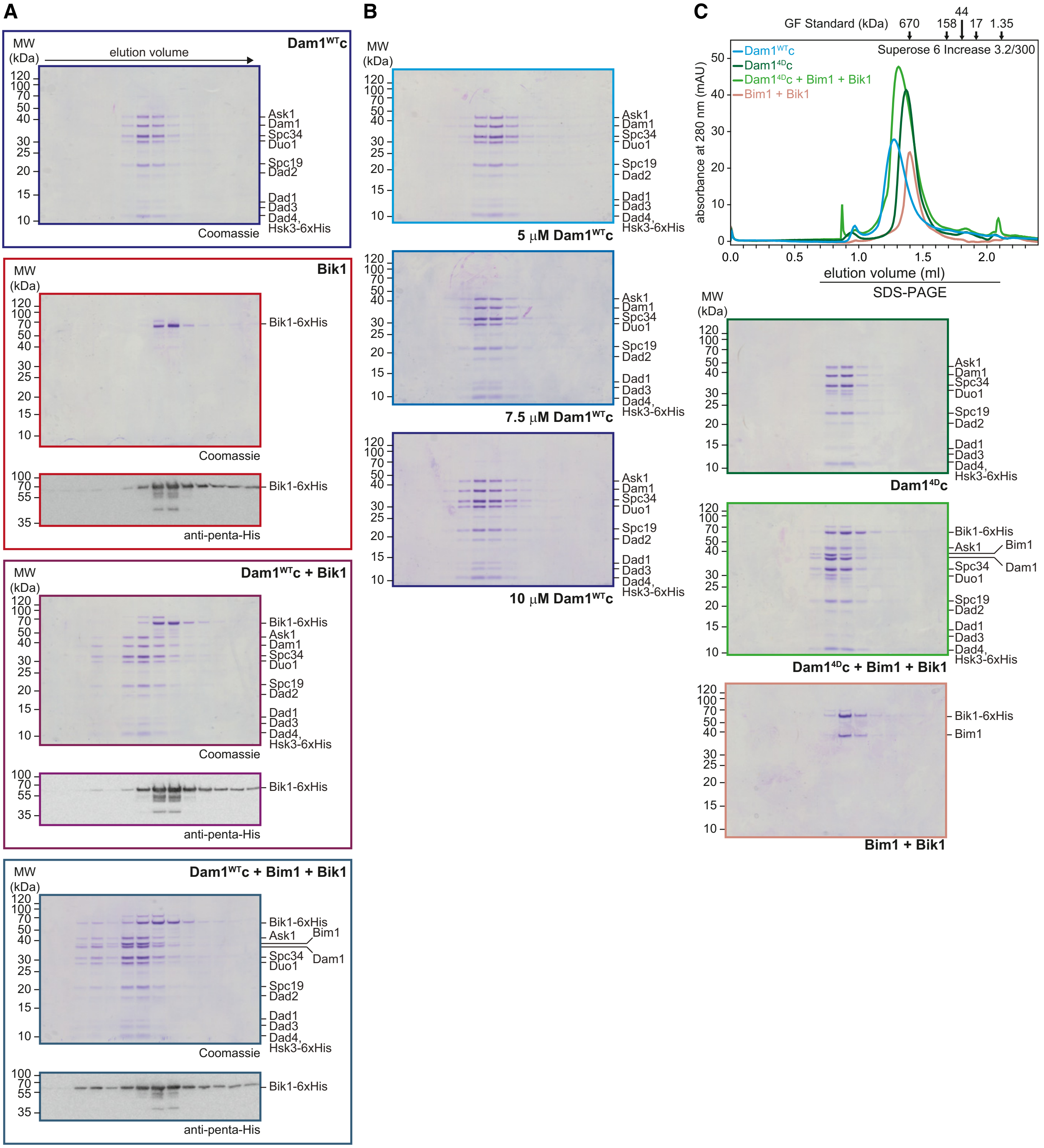

Figure EV4. Detailed analysis of Bik1 binding to Dam1c.

A Western blot analysis of elution fractions from size-exclusion chromatography of Dam1^{WT}c with Bim1 and Bik1. Bik1-6xHis was detected using an anti-penta-His antibody. Western blots are shown together with the corresponding Coomassie-stained gels.

B SDS–PAGE analysis of size-exclusion chromatography of Dam1c with different input concentrations. Corresponding chromatograms are shown in Fig 5A.

C Analytical size-exclusion chromatography of Dam1^{4D}c alone and in combination with Bim1-Bik1. Chromatograms and SDS–PAGE analysis of elution fractions are shown. In addition, the chromatogram of Dam1^{WT}c is shown to emphasize the different retention volume compared with Dam1^{4D}c.

 © 2021 The Authors

Figure EV5. Biochemical analysis of Ndc80c binding to pre-assembled Dam1c-Bim1-Bik1 complex.

A Recombinant Ndc80c and Bim1 individually or in combination were analyzed by analytical size-exclusion chromatography. Chromatograms and SDS–PAGE analysis of elution fractions are shown. The 3xFlag peptide used for elution of Ndc80c during purification was eluted after 2 ml and caused an increase in the absorbance at 280 nm.

B, C Analytical size-exclusion chromatography to analyze binding of Ndc80c to Dam1c-Bim1-Bik1 complex. Proteins alone or in combination were subjected to size-exclusion chromatography using a running buffer with 150 mM NaCl. Chromatograms are shown in (B), and SDS–PAGE analysis of elution fractions is shown in (C). Subunits of Dam1c and Ndc80c are labeled in blue and red, respectively, and Bim1 and Bik1 in black. In (B), an additional magnification of the chromatogram is depicted for better visualization of relatively low absorbance peaks.

© 2021 The Authors

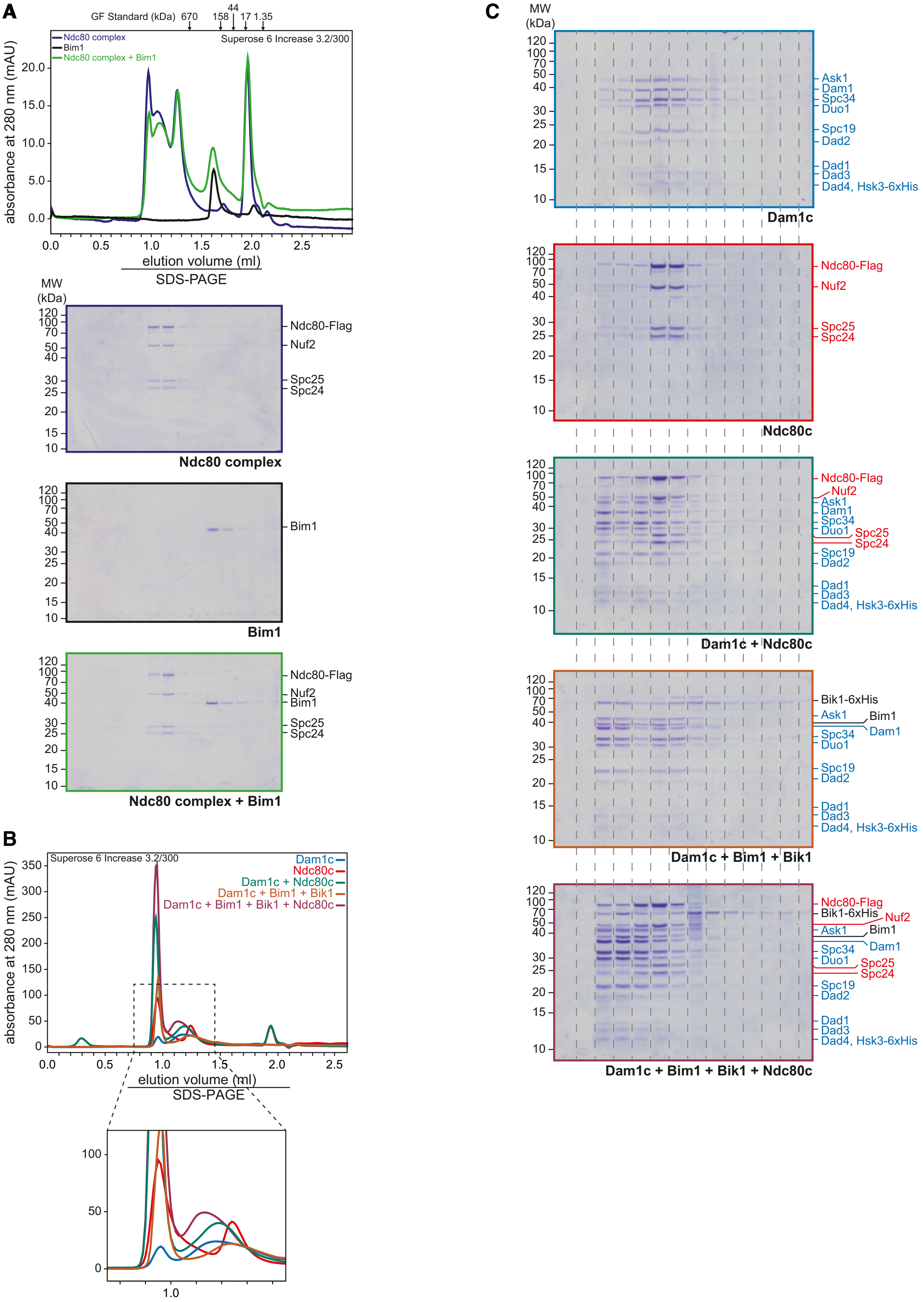

Figure EV5.

© 2021 The Authors
